# Intraluminal Endovascular Coil Migration: A Rare Complication Post-Embolization of the Gastroduodenal Artery for a Previously Bleeding Duodenal Ulcer

**DOI:** 10.7759/cureus.14615

**Published:** 2021-04-21

**Authors:** Yassin Naga, Mahendran Jayaraj, Yousif Elmofti, Annie Hong, Gordon Ohning

**Affiliations:** 1 Internal Medicine, University of Nevada Las Vegas School of Medicine, Las Vegas, USA; 2 Gastroenterology, University of Nevada Las Vegas School of Medicine, Las Vegas, USA

**Keywords:** upper gastrointestinal bleed, coil migration, upper endoscopy, duodenal ulcer, esophagogastroduodenoscopy (egd)

## Abstract

Transarterial angiographic embolization is a highly effective, safe treatment for non-variceal upper gastrointestinal bleeding refractory to endoscopic intervention. However, intraluminal coil migration is a possible complication. Coil migration, while usually a self-limiting process, can lead to significant rebleeding. In our case, a patient presented with a life-threatening duodenal ulcer hemorrhage, likely precipitated by intraluminal endovascular coil migration after a recent gastro-duodenal artery embolization. He was successfully managed without endoscopic coil removal and had no additional gastrointestinal bleeding. It is important for endoscopists to be aware of this complication and weigh the risks and benefits of coil removal.

## Introduction

Approximately 8%-15% of patients with a non-variceal upper gastrointestinal bleed (UGIB) have persistent bleeding despite endoscopic hemostatic intervention [1]. Transarterial angiographic embolization (TAE) plays an important role when endoscopic interventions have failed. It has also decreased the need for salvage surgery [2]. TAE has been shown to have high clinical and technical success rates [3]. Coil embolization specifically is a safe intervention with a low rate of complications, which includes perforation, artery dissection, and coil migration [3-4]. In most cases, coil migration does not lead to severe complications although it can cause rebleeding and bowel ischemia [5-7]. Herein, we report a rare case of spontaneous intraduodenal coil migration and excretion following a recent coil embolization due to a bleeding duodenal ulcer nine days prior to admission.

## Case presentation

**Figure 1:**
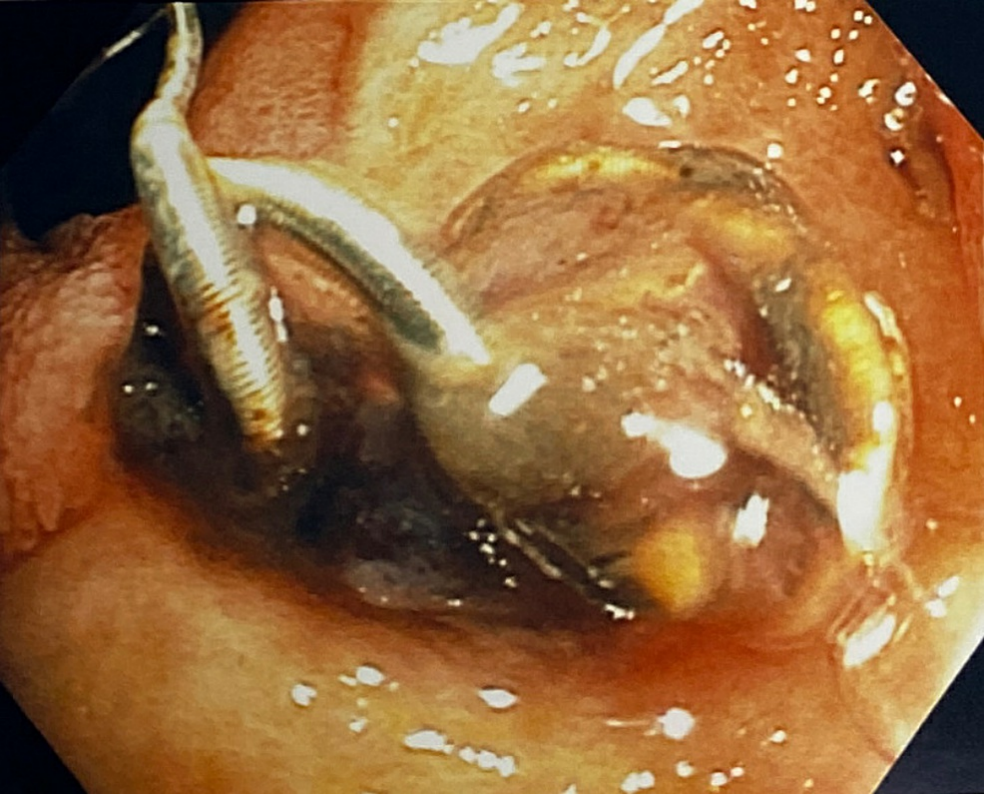
Esophagogastroduodenoscopy image of an unattached metal coil in the distal duodenal bulb ulcer

**Figure 2:**
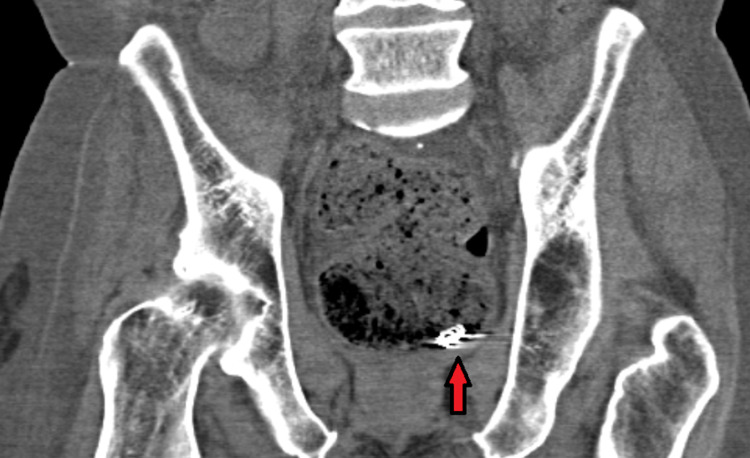
High-density focus in the rectal lumen suspected to be the migrated endovascular coil

A 68-year-old male with a history of recent hospitalization due to duodenal ulcer bleeding presented to the hospital in cardiac arrest. Cardiopulmonary resuscitation led to a return of spontaneous circulation. During intubation, blood was observed in the oropharynx and a large amount of bright red blood was noted upon placement of an orogastric tube. He was persistently hypotensive and required aggressive resuscitation with three units of packed red blood cells for initial hemoglobin of 5.2g/dL. He underwent an urgent esophagogastroduodenoscopy (EGD), which demonstrated multiple, large, conjoined duodenal ulcers in a deformed duodenal bulb that extended into the descending duodenum. A profusely bleeding vessel was seen at the base of the proximal duodenal bulb ulcer (Forrest Class 1A). In addition, a luminal metal coil with an overlying clot and debris was visualized at the base of a more distal duodenal bulb ulcer that extended into the descending duodenum (Figure 1). Successful endoscopic hemostasis was achieved with epinephrine injection and multipolar electro-cautery application. A hemoclip was placed on the visible vessel to mark the location for the radiologist. The exposed metal coil was left in place, as the immediate pressing matter was hemostasis. Due to the likely involvement of the gastroduodenal artery and the high risk of rebleeding, Interventional Radiology was consulted. An angiogram was performed, and no evidence of active extravasation was identified. However, multiple small left gastric artery branches appeared to feed the previously bleeding proximal duodenal bulb ulcer (Forrest Class 1A) identified by the presence of a hemoclip. Detachable embolization coils were placed in two branches of the left gastric artery. The angiogram also demonstrated an existing embolization coil in the gastroduodenal artery with no distal flow. A review of medical records revealed that this was the metal coil deployed for a UGIB at an outside hospital nine days prior. This was the same metal coil seen during endoscopy after it extruded through the duodenal wall. A follow-up abdominal CT obtained four days after hemostasis was achieved for an evaluation of shock, which demonstrated a high-density metallic focus in the lumen of the rectum with significant stool burden (Figure 2). He did not experience recurrent episodes of UGIB prior to discharge.

## Discussion

UGIB remains an important clinical challenge that carries significant morbidity and mortality rates. Endoscopic hemostatic intervention is the recommended first-line treatment [8]. Surgery and TAE are alternative therapies in patients with refractory bleeding despite endoscopic intervention. TAE is a highly effective intervention, as it allows for both diagnostic and therapeutic intervention. If the ulcer overlies the posterior duodenal bulb or lesser curvature of the stomach, it can lead to erosion of the gastroduodenal artery and left gastric artery, respectively. Our patient presented in cardiac arrest with hypovolemic shock due to a bleeding ulcer located at the posterior bulbar duodenum. He had undergone coil embolization of the gastroduodenal artery for another duodenal ulcer nine days prior. Multiple left gastric artery branches were responsible for this current GI bleed and were subsequently embolized after endoscopic hemostasis.

The overall technical and clinical success of TAE for non-variceal UGIB was reported in several studies as 92%-100% and 51%-94%, respectively [9-12]. When compared to surgical intervention, TAE resulted in shorter hospital durations, fewer complications, and lower 30-day mortality [13-14]. Therefore, TAE is considered the first-line therapy for non-variceal UGIB refractory to endoscopic intervention. Several potential complications can arise from TAE, including access site hematomas (3%-17%), pseudoaneurysm, and coil migration (3%) [15-17].

Intraluminal coil migration is a rare but notable complication of TAE that can occur immediately or several years later [16]. It is hypothesized that embolization of the feeding artery leads to ischemia, which allows for coil migration through the bowel wall [16]. In our patient, a displaced coil was seen on endoscopy at the time of intervention. He was subsequently found to have a metallic focus in the rectum on a CT abdomen obtained four days later, indicating coil detachment from the duodenal ulcer base and intraluminal migration out through the rectum. In most cases, coil migration is a self-limiting process without significant complications. However, it can lead to severe complications, including fatal rebleeding and bowel obstruction [16-18]. It is unclear whether our patient had rebleeding from coil migration itself or a separate ulcer invading the left gastric artery branches, but the onset of the bleed shortly after the TAE procedure and the finding of an embolization coil in the lumen within close proximity of the active bleeding suggests that migration of the coil was the precipitating event. In addition, CPR may have further displaced the migrated coil away from the bleeding ulcer base. The current management for migrated endovascular coils remains unclear. Several cases reported that migrated coils have spontaneously migrated through the rectum without any intervention, although this carries the risk of mucosal damage and rebleeding [4,15]. On the other hand, several cases have also reported on the successful removal of the migrated coils; however, bleeding remains a major risk that needs to be considered [18-20].

## Conclusions

In conclusion, we report a rare case of a life-threatening recurrent UGIB caused by spontaneous intraluminal coil migration following recent gastroduodenal artery coil embolization. Although rare, it is an important complication that clinicians should be aware of. Currently, the management of migrated coils remains unclear and more research is necessary to help formulate guidelines.
